# Carbon Nanotube-Silicon Nanowire Heterojunction Solar Cells with Gas-Dependent Photovoltaic Performances and Their Application in Self-Powered NO_2_ Detecting

**DOI:** 10.1186/s11671-016-1514-6

**Published:** 2016-06-14

**Authors:** Yi Jia, Zexia Zhang, Lin Xiao, Ruitao Lv

**Affiliations:** Qian Xuesen Laboratory of Space Technology, China Academy of Space Technology, Beijing, 100094 China; Key Laboratory of Advanced Materials (MOE), School of Materials Science and Engineering, Tsinghua University, Beijing, 100084 China

**Keywords:** Carbon nanotube, Silicon nanowire, Heterojunction, Solar cell, Gas sensor

## Abstract

A multifunctional device combining photovoltaic conversion and toxic gas sensitivity is reported. In this device, carbon nanotube (CNT) membranes are used to cover onto silicon nanowire (SiNW) arrays to form heterojunction. The porous structure and large specific surface area in the heterojunction structure are both benefits for gas adsorption. In virtue of these merits, gas doping is a feasible method to improve cell’s performance and the device can also work as a self-powered gas sensor beyond a solar cell. It shows a significant improvement in cell efficiency (more than 200 times) after NO_2_ molecules doping (device working as a solar cell) and a fast, reversible response property for NO_2_ detection (device working as a gas sensor). Such multifunctional CNT-SiNW structure can be expected to open a new avenue for developing self-powered, efficient toxic gas-sensing devices in the future.

**Electronic supplementary material:**

The online version of this article (doi:10.1186/s11671-016-1514-6) contains supplementary material, which is available to authorized users.

## Background

There is a growing requirement of lightweight, energy-saving, integrated devices for various wearable and portable electronic applications. In particular, multifunctional devices combining energy generation and other specific function are highly desired in many applications. To this end, functionalized piezotronic generators and solar cells with additional properties, such as wind speed [1], UV [2] and pressure [3] detecting, have been developed accordingly. In recent years, carbon nanotube (CNT)-silicon heterojunction structure has been considered as one of facile and promising designs for many applications [4–6]. In this structure, CNT films are assembled with silicon wafer to form heterojunction, in which the built-in electric field is generated and driving charge carriers to electrodes. Particularly, owing to its potential prospect for developing high-efficiency and low-cost solar cells, the heterojunction structure has attracted great research interest in both elucidating its working mechanism [7, 8] and improving its power conversion efficiency (*η*) [9–15]. Some key points in this structure are crucial to the junctions’ performance, including properties of carbon materials (sheet resistance, light transmissivity, etc.), substrate wafer (resistance, energy band gap, light transmissivity, etc.), and their interface (contact state, thickness). To improve the cell efficiency, different strategies focusing on those points have been proposed accordingly. Firstly, chemical doping with volatile oxidants [9–11, 14] was applied to CNT membranes to adjust their Fermi level and carrier concentration. Secondly, an insulator layer (SiO_x_) with a suitable thickness was introduced at the interface of CNT membrane and silicon wafer to form a metal-insulator-semiconductor (MIS) junction [11, 15]. The existence of this insulator layer significantly suppressed carrier recombination and improved the diode ideality factor of CNT-Si junction. Moreover, surface anti-reflection treatments (PDMS [11], TiO_2_ [12], and MoO_x_ [13] coating) were also efficient to enhance the light absorption of CNT-Si devices. In addition, silicon nanowire (SiNW) arrays with their unique one-dimensional aligned structure and outstanding electrical properties have exhibited excellent light trapping and carrier-transporting performances [16]. In our previous work, SiNW arrays have been used to assembly heterojunction solar cells with CNT membranes. Electrolyte was also used to fill into the pores between SiNW arrays and CNT membranes to provide additional channels for charge transport [17]. Thus, the cell efficiency increased from 0.092 to 1.29 % after electrolyte infiltration. However, without extra-encapsulation, cell stability was poor due to the gradual evaporation of electrolyte.

In this work, we utilize SiNW arrays to fabricate heterojunction with CNT membranes for reducing light reflection, followed by NO_2_ gas doping to increase cell performances. In this structure, the large specific surface area of both SiNW arrays and CNT membranes, together with additional channels built by their point-to-line contact, can greatly facilitate gas adsorption and desorption, which will further improve cell performances. To take full advantages of this structure, we also demonstrate the gas-sensitive properties of CNT-SiNW heterojunction structure. Compared with traditional metallic oxide gas sensors working at relatively high temperatures, this CNT-SiNW gas sensor works at room temperature. Particularly, it combines the functions of solar cell and gas sensor, as a result, this CNT-SiNW gas sensor is self-powered (by light), which is more energy-efficient and safer, especially for explosive gases detecting.

## Methods

### Chemical Etching for SiNW Arrays

SiNW arrays used in this study were prepared by a Ag-assisted etching method. *n*-type (100) silicon wafers with the electrical resistivity of 2~4 Ω cm were cleaned with acetone, ethanol, and piranha solution (H_2_O_2_ and H_2_SO_4_), followed immersing into HF and AgNO_3_ mixture solution for 15 min to fabricate SiNW arrays. After that, the as-prepared SiNW arrays were rinsed in deionized water and treated with HF and HNO_3_ to remove dendrite silver films covered onto SiNW arrays. The height of SiNWs was 300 nm. Then a Ti/Au layer (50 nm) was deposited on the back side of SiNW arrays.

### Synthesis of CNT Membranes

High-quality CNT membranes were synthesized by a chemical vapor deposition (CVD) method using xylene as carbon source, ferrocene, and sulfur as catalyst precursor, respectively. The reaction temperature was set at 1160 °C, and CNT membranes were collected onto a piece of nickel foil at the downstream of quartz tube reactor.

### Assembly and Test of CNT-SiNW Solar Cells

The as-prepared spiderweb-like CNT membranes were directly lifted up and transferred onto SiNW arrays to construct CNT-SiNW heterojuction devices. The active area for each solar cell was 0.24 cm^2^. In order to investigate the effects of NO_2_ modification on cell performances, CNT-SiNW solar cells were sealed into a quartz chamber with a window for light illumination from a solar simulator. The light intensity in the quartz chamber was 80 mW/cm^2^, which was calibrated by a silicon solar cell. The temperature in the quartz chamber was kept at room temperature. The flow rates of NO_2_ and N_2_ in the quartz chamber were adjusted by two mass flow controllers, respectively. Thus, the NO_2_ concentration could be finely controlled from 0 to 1000 ppm. The *I*-*V* data of CNT-SiNW solar cells were recorded by a Keithley 2601 digital source-meter.

### Test of CNT-SiNW Gas-Sensing Properties

To test gas-sensing properties, CNT-SiNW solar cells were sealed into the same quartz chamber with some appropriate changes in the devices and equipment. First, a cold light source (LED) was used here to replace the solar simulator. Thus, the thermal effect of solar light source on cell performance could be eliminated. Second, SiNW arrays used to fabricate solar cell here had been stored in air for more than half a year to form a stable interfacial oxide layer. In this case, the non-reversible interfacial oxide layer effect could be avoided during gas-sensitive testing process. Third, the exhaust gas of the quartz chamber was evacuated by a vacuum pump. The *V*-*t* data of CNT-SiNW gas sensors were also recorded by the Keithley 2601 digital source-meter.

## Results and Discussion

**Fig. 1:**
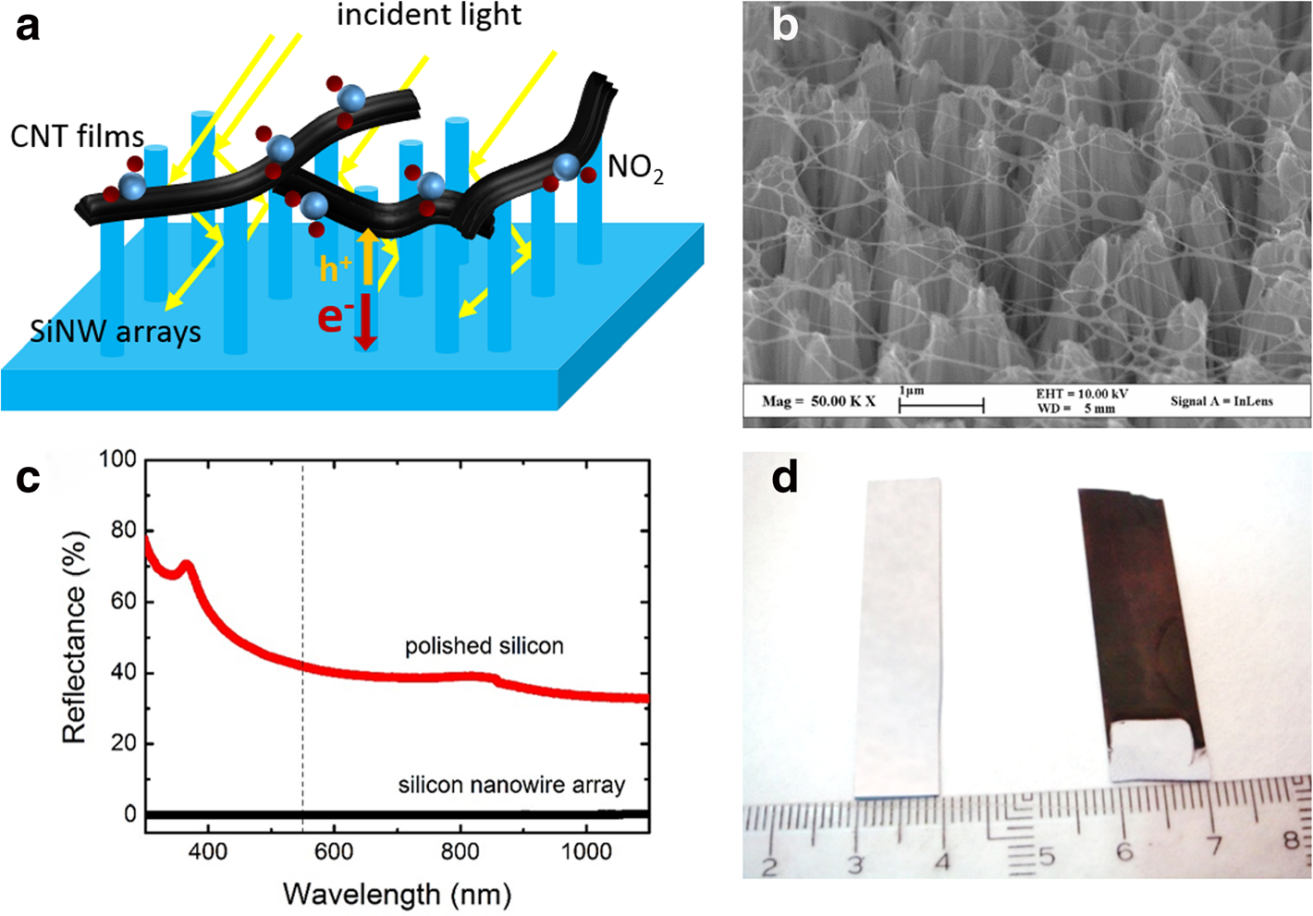
Characterization of CNT-SiNW solar cells. **a** Schematic diagram of the CNT-SiNW solar cells. **b** SEM images of CNT-SiNW heterostructures. **c** Reflection spectra of polished silicon wafer and SiNW arrays. **d** Photographs of polished silicon wafer (*left*) and SiNW arrays (*right*)

Figure 1a shows that the heterojunction consists of an *n*-type SiNW array wafer coated by a semi-transparent CNT membrane. Incident photons are absorbed by vertical SiNWs and converted into photon-generated carriers. Those charge carriers are directly separated by the built-in field at the CNT-Si junction and then driven to CNT membranes (for hole) and SiNW array wafer (for electrons) at the same time. NO_2_ molecules with controlled concentration are injected and adsorbed onto the heterojunction surfaces to improve cell performances. SEM image in Fig. 1b reveals that CNT membranes are uniformly coated onto SiNW arrays to form a heterojunction for the separation and transport of charge carriers. It also shows thousands of traps in SiNW arrays; thus, light reflection at their surface is remarkably suppressed. The optical reflection spectra of SiNW arrays and polished silicon wafer are shown in Fig. 1c. The polished silicon wafer shows a lager reflectance about 40~80 % from near-infrared to ultraviolet region. After chemical etching, the reflectance of SiNW array surface sharply decreases to nearly 0 %. Accordingly, in the photograph of Fig. 1d, the mirror-like silicon wafer converts to ultra-black SiNW arrays after chemical etching.

**Fig. 2:**
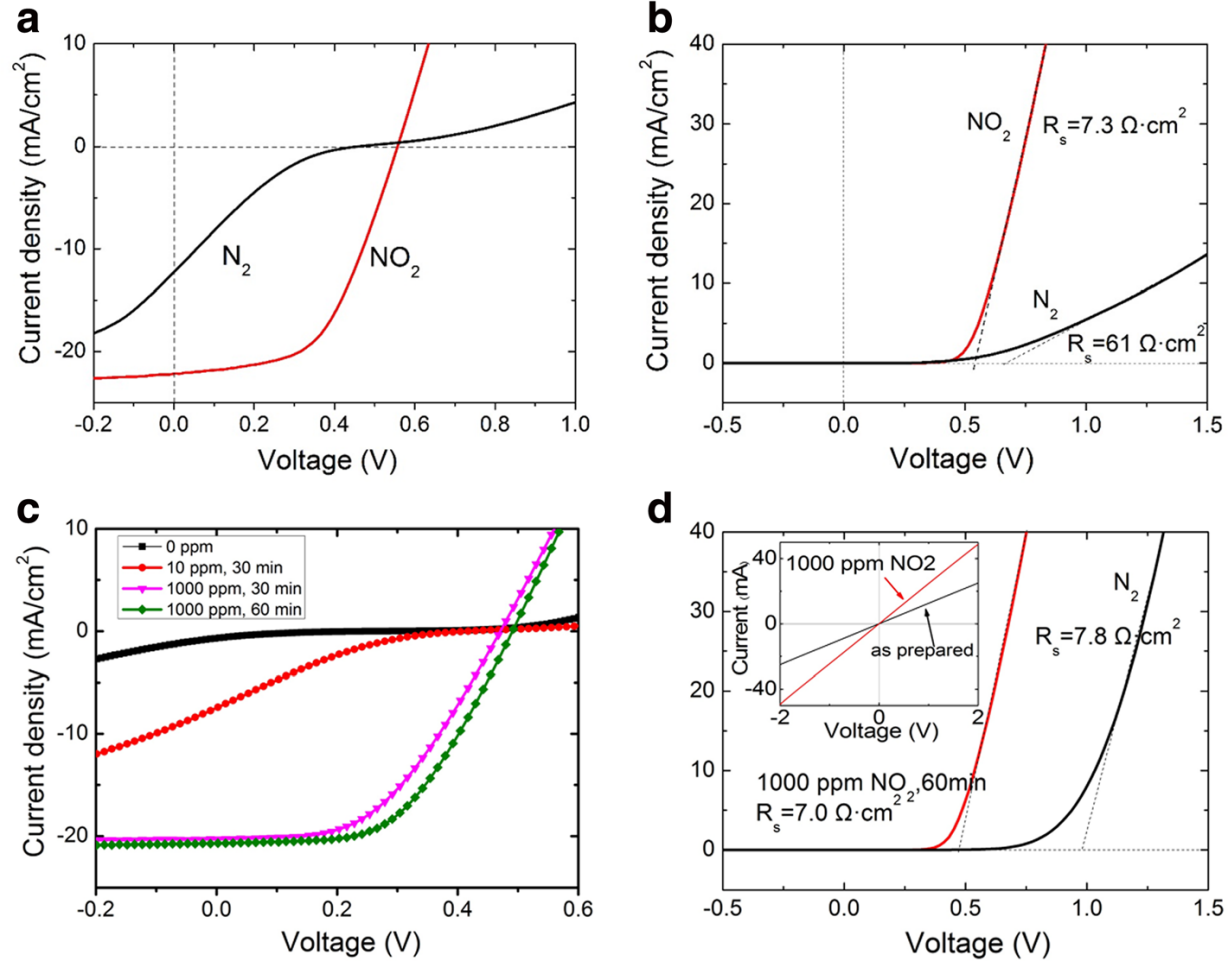
Effect of NO_2_ doping on CNT-SiNW solar cell performances. **a** Light *J*-*V* curves of CNT-SiNW solar cell before (*black*) and after (*red*) NO_2_ doping. **b** Dark *J*-*V* curves of CNT-SiNW solar cell before (*black*) and after (*red*) NO_2_ doping. **c** Light *J*-*V* curves of CNT-SiNW solar cell at different NO_2_ concentration and exposure time. **d** Series resistance decrease after 1000 ppm NO_2_ doping for 60 min. *Inset* is the *I*-*V* curves of a CNT membrane before and after NO_2_ doping

The porous structures of both CNT membranes and SiNW arrays can greatly facilitate gas adsorption and desorption, which can influence cell performances. In Fig. 2a, the current density versus voltage (*J*-*V*) curves are shown for pristine and NO_2_-doped CNT-SiNW solar cells under light illumination. The pristine CNT-SiNW solar cell (with a thin native oxide layer) shows a *V*_oc_ = 0.45 V, *J*_sc_ = 12.1 mA/cm^2^, FF = 17 %, and a cell efficiency (*ƞ*) of 1.2 %. After NO_2_ doping, those performances increase to *V*_oc_ = 0.55 V, *J*_sc_ = 22.2 mA/cm^2^, FF = 54.4 %, and *ƞ* = 8.4 %. Corresponding dark *J*-*V* curves in Fig. 2b reveal that the series resistance (*R*_s_) drops from 61 to 7.3 Ω cm^2^ and the rectification ration at ±0.75 V increases from 3000 to 50,000. Those results from dark *J*-*V* curves indicate an improvement in diode characteristics for CNT-SiNW solar cell after NO_2_ modification. Next, the influences of different NO_2_ concentration and treating time on cell performance were studied. Figure 2c shows a series of dramatic shifts in *J*-*V* curves when a solar cell was exposed in different NO_2_ conditions (the characteristics are summarized in Table 1. The fresh cell (cell surface was rinsed by dilute HF solution and deionized water) shows a *V*_oc_ = 0.28 V, *J*_sc_ = 0.66 mA/cm^2^, a poor FF = 12.5 %, and a power conversion efficiency of 0.03 %. With a small amounts of NO_2_ treatment (10 ppm, 30 min), there are significant improvements in *V*_oc_ (0.42 V) and *J*_sc_ (7.4 mA/cm^2^). After 1000 ppm NO_2_ treatment for 60 min, the cell performances reached a *V*_oc_ = 0.48 V, *J*_sc_ = 20.7 mA/cm^2^, FF = 54.3 %, and *ƞ* = 6.74 %. The final cell efficiency is more than 200 times of the initial *ƞ*. Previous studies have demonstrated that a thin oxide layer at the CNT-Si cell could result in significant *ƞ* enhancement [8, 11, 15]. Obviously, the oxidation ability of NO_2_ is much stronger than that of air. Thus, the formation of interface oxide layer (Additional file 1: Figure S1) could be one reason for cell performance improvement. Besides that, NO_2_ treatment not only affected on the interface state of CNT-Si heterojunction but also can remarkably decrease the resistance of the device. The inset in Fig. 2d shows that CNT membrane sheet resistance reduces from 79.7 to 40.5 Ω/□ after keeping in NO_2_ atmosphere for 60 min. The reduction of the sheet resistance further leads to the change of series resistance (*R*_s_) for solar cells. The *R*_s_ of solar cell in N_2_ and NO_2_ treatment states have been calculated from corresponding dark *J*-*V* curves. Consistent with the CNT membrane sheet resistance reduction, after 1000 ppm NO_2_ doping for 60 min, the solar cell’s series resistances decreases from 7.8 to 7.0 Ω cm^2^, as shown in Fig. 2d.

**Table 1:** Characteristics of the CNT-SiNW solar cells under AM1.5G, 80 mW/cm^2^ illumination treated at different NO_2_ concentrations and exposure time

Device treatment	*V*_oc_(V)	*J*_sc_(mA/cm^2^)	FF (%)	*η* (%)
0 ppm (N_2_)	0.28	0.66	12.5	0.03
NO_2_ (10 ppm, 30 min)	0.42	7.40	16.9	0.65
NO_2_ (1000 ppm, 30 min)	0.47	20.3	48.9	5.80
NO_2_ (1000 ppm, 60 min)	0.48	20.7	54.3	6.74

**Fig. 3:**
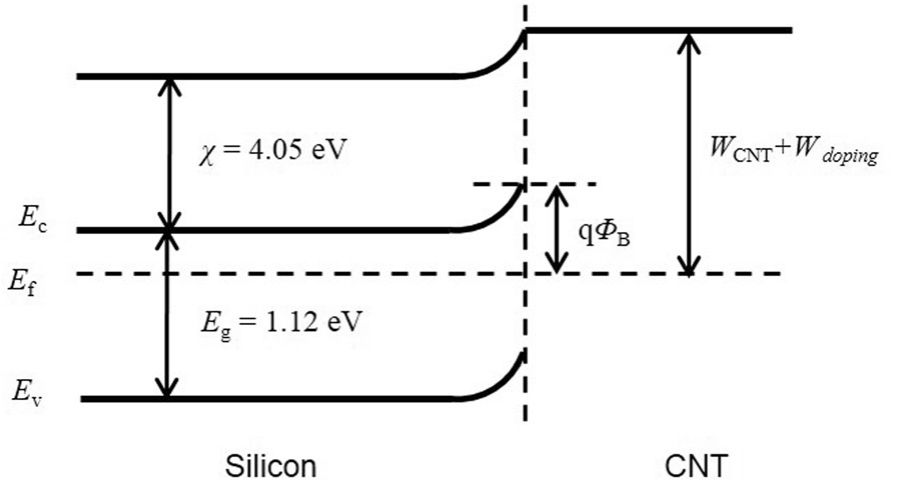
Energy band diagram of CNT-SiNW heterojunction

Based on previous reports, the CNT-Si structure can be considered as a Schottky junction, in which CNT membrane serves as metallic materials while silicon wafer serves as a semiconductor [8, 11, 15]. Figure 3 is the corresponding energy band diagram for CNT-SiNW junction. In this diagram, the barrier height (*Φ*_B_) is equal to the work function difference between CNT membranes and silicon nanowire; the equation for the barrier height [18] is

1 $$ q{\varPhi}_{\mathrm{B}}=\left({W}_{\mathrm{CNT}}+{W}_{\mathrm{doping}}\right)-\chi $$

where *q* is the electron charge, *Φ*_B_ is the barrier height, *W*_CNT_ is the work function of CNT membrane (4.8 eV), *W*_doping_ is the additional doping work function of CNT membrane, and *χ* is electron affinity of silicon (4.05 eV). For CNT-SiNW heterojunction junction in Fig. 3, when NO_2_ molecules modified onto CNT membranes surface, the work function of CNT membranes was up-shifted, leading to the increases of *Φ*_B_ in Eq. (1) and energy diagram.

According to theories for Schottky junction solar cells, the *V*_oc_ can be expressed as [18]:

2 $$ {V}_{\mathrm{oc}}=n\left[q{\varPhi}_{\mathrm{B}}+\left(kT/\mathrm{q}\right)1\mathrm{n}\left({I}_{\mathrm{s}}/{A}_e{A}^{\ast }{T}^2\right)\right] $$

where *n* is the diode ideality factor, *k* is the Boltzmann constant, *T* is the working temperature, *I*_s_ is the diode saturation current, and *A*_e_ and A^*^ are the contact area of the diode and the Richardson constant, respectively. In Eq. (2), *V*_oc_ is positively correlated with *Φ*_B_. After NO_2_ molecules doping, the up-shift of CNT work function and increase of *Φ*_B_ led to the improvements of *V*_oc_ for solar cells, as shown in Fig. 2.

**Fig. 4:**
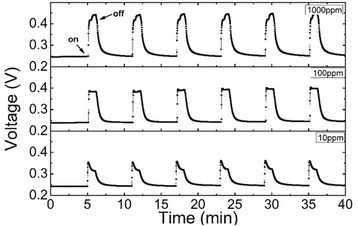
Real-time NO_2_ detection with different concentrations using CNT-SiNW gas sensor

The results above have demonstrated that NO_2_ gas can modify both CNT membranes and heterojunction interface to improve cell performance. On the other hand, from the energy band diagram in Fig. 3, it also means that the cell performance is sensitive to the ambient environment. Moreover, the adsorption site for NO_2_ molecules onto CNTs is the top of a carbon atom and the charge transfer is −0.06~−0.09 electron per molecule, resulting in a weak binding (0.4~0.8 eV) between them [19]. The non-covalent combination leads to a reversible behavior in cell performance by switching NO_2_ molecule adsorption and desorption states. Starting from this point, we used a quartz chamber to test CNT-SiNW junctions’ gas-sensing properties. Figure 4 is the real-time NO_2_ detection by CNT-SiNW heterojunction. In vacuum condition, the *V*_oc_ of the device is 0.24 V. When the device is exposed in small amounts of NO_2_ gas (e.g., 10 ppm), a fast response of *V*_oc_ can be achieved in 4~6 s, the *V*_oc_ sharply increases to 0.35 V, which is improved more than 40 % compared with that of the initial untreated devices. After switching the NO_2_ concentration from 0 to 10 ppm for six times, the maximum response remains unchanged, indicating a good repeatability of this CNT-SiNW heterojunction sensor. Further increasing the concentration to 100 ppm and 1000 ppm, the *V*_oc_ will increase to 0.39 and 0.44 V, respectively.

**Fig. 5:**
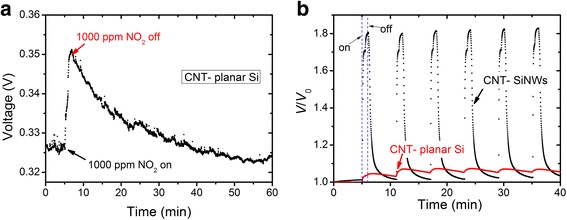
CNT-planar silicon structure responses to 1000 ppm NO_2_ exposure. **a** One response and recovery process of CNT-planar silicon structure. **b** Normalized voltage responses of CNT-SiNWs and CNT-planar silicon under repeated exposures of 1000 ppm NO_2_ at room temperature

To study the effect of CNT-SiNW structure on gas detection, a control sample of CNT-planar silicon was tested under repeated exposures of 1000 ppm NO_2_ at room temperature. The result in Fig. 5a shows that it needs more than 40 min for 1000 ppm NO_2_ desorption from CNT-planar silicon sample, which is much longer than the recovery time in CNT-SiNWs (less than 5 min). Moreover, Fig. 5b also shows that the normalized voltage responses of CNT-SiNW structure (~1.8) are much larger than the responses of CNT-planar silicon (~1.1) under repeated exposures of 1000 ppm NO_2_. All the results above indicate that CNT-SiNW structure plays an important role in improving device’s response speed and amplitude.

## Conclusions

In summary, we directly assembled CNT membranes with SiNW wafer to form heterojunction for solar cells and gas sensors. The CNT-SiNW heterojunction showed a gas-dependent photovoltaic effect. Thus, the power conversion efficiencies of CNT-SiNW solar cells are up to 8.4 % after NO_2_ gas doping. The CNT-SiNW heterojunction also demonstrated a self-powered gas detection sensitivity at room temperature. This CNT-SiNW heterojunction-based gas sensor will lead to much more sensitive and simple carbon-based gas sensors in the future.

## Additional file

Additional file 1: Figure S1. Depth-resolved Auger spectra of silicon and oxygen atomic concentrations in a fresh Si substrate and after 1000 ppm NO_2_ oxidation. (TIF 7878 kb)
